# Identifying Differences in Molecular Characteristics Relevant for Remodeling of Periodontal Ligament Stem Cells from the Upper and Lower Jaw

**DOI:** 10.3390/ijms25063207

**Published:** 2024-03-11

**Authors:** Hanna Malyaran, Rogerio B. Craveiro, Sinan Mert, Christian Niederau, Sanne L. Maas, Emiel P. C. van der Vorst, Frank Hölzle, Wilhelm Jahnen-Dechent, Michael Wolf, Sabine Neuss

**Affiliations:** 1 Interdisciplinary Center for Clinical Research (IZKF), RWTH Aachen University, 52074 Aachen, Germany; hmalyaran@ukaachen.de (H.M.); smaas@ukaachen.de (S.L.M.); evandervorst@ukaachen.de (E.P.C.v.d.V.); 2 BioInterface Group, Helmholtz Institute for Biomedical Engineering, RWTH Aachen University, 52056 Aachen, Germany; willi.jahnen@rwth-aachen.de; 3 Department of Orthodontics, University Hospital of RWTH Aachen, 52074 Aachen, Germany; rcraveiro@ukaachen.de (R.B.C.); cniederau@ukaachen.de (C.N.); michwolf@ukaachen.de (M.W.); 4 Division of Hand, Plastic and Aesthetic Surgery, LMU University Hospital Munich, 80336 Munich, Germany; sinan.mert@rwth-aachen.de; 5 Institute for Molecular Cardiovascular Research (IMCAR), RWTH Aachen University, 52074 Aachen, Germany; 6 Aachen-Maastricht Institute for CardioRenal Disease (AMICARE), RWTH Aachen University, 52074 Aachen, Germany; 7 Institute for Cardiovascular Prevention (IPEK), Ludwig-Maximilians-University Munich, 80336 Munich, Germany; 8 Department of Oral and Maxillofacial Surgery, University Hospital of RWTH Aachen, 52074 Aachen, Germany; fhoelzle@ukaachen.de; 9 Institute of Pathology, RWTH Aachen University, 52074 Aachen, Germany

**Keywords:** kinase activity, periodontal ligament stem cells, mandible, maxilla, tyrosine, serine/threonine

## Abstract

Periodontal defects’ localization affects wound healing and bone remodeling, with faster healing in the upper jaw compared to the lower jaw. While differences in blood supply, innervation, and odontogenesis contribute, cell-intrinsic variances may exist. Few studies explored cell signaling in periodontal ligament stem cells (PDLSC), overlooking mandible-maxilla disparitiesUsing kinomics technology, we investigated molecular variances in PDLSC. Characterization involved stem cell surface markers, proliferation, and differentiation capacities. Kinase activity was analyzed via multiplex kinase profiling, mapping differential activity in known gene regulatory networks. Upstream kinase analysis identified stronger EphA receptor expression in the mandible, potentially inhibiting osteogenic differentiation. The PI3K-Akt pathway showed higher activity in lower-jaw PDLSC. PDLSC from the upper jaw exhibit superior proliferation and differentiation capabilities. Differential activation of gene regulatory pathways in upper vs. lower-jaw PDLSC suggests implications for regenerative therapies.

## 1. Introduction

Stem cell biology is of great importance for tissue regeneration and applications in Tissue Engineering and Regenerative Medicine. Mesenchymal stem cell (MSC)-like populations from various tissues are defined using the ‘gold standard’ minimal criteria of the International Society for Cellular Therapy, which were first elaborated in bone marrow MSC 1968 [1]. Stem cells possess anti-inflammatory properties, encourage the proliferation of epithelial cells, and hinder wound scarring [2]. Moreover, both stem cells and their exosomes exert notable impacts on the regeneration of bone tissue and the nervous system [3,4]. Different cell types with stem cell properties have been derived and characterized from different dental/oral tissues, making the teeth and the supporting oral tissues attractive and accessible sources of stem cells [5,6,7]. The differentiation mechanisms and efficacy of dental stem cells in bone tissue regenerative medicine have been extensively investigated [8]. These stem cells have the ability to differentiate into osteoblasts, chondroblasts, and adipocytes [9,10]. Since 2004, periodontal ligament (PDL) cells have been isolated from extracted third molars, displaying self-renewal and differentiation potential towards multiple mesodermal cell fates, and thus are referred to as periodontal ligament stem cells (PDLSC). As a component of the periodontal apparatus, they serve as a crucial regulator of the microenvironment necessary for tooth anchoring and feeding, as well as functions of proprioception, action force buffering, periodontal remodeling, and mechanical signal transmission. These cells can be implanted into periodontal deficiencies to improve periodontal regeneration [11]. PDLSC have been considered ideal candidates for periodontal regeneration, since they can be easily isolated by non-invasive procedures after simple tooth extraction. Stem cells from different sources have different molecular and growth characteristics; therefore, stem cell-mediated repair and tissue regeneration mechanisms and effects may differ [12].

Clinical experience holds that the precise localization of periodontal defects greatly influences the velocity and effectiveness of wound healing and bone remodeling. Periodontal healing is quicker and more efficient in the maxilla (upper jaw) than in the mandible (lower jaw). Differences in blood supply, innervation, and oncogenic development/odontogenesis of upper vs. lower jaw may all influence healing [13]. In addition, the composition of alveolar bone in the mandible and maxilla differs: the mandible contains 63% lamellar bone and 16% bone marrow, while the maxilla includes around 46% lamellar bone and 23% bone marrow. While the mandible is primarily composed of dense cortical bone that may be grafted, the maxilla is made up of porous cancellous bone that resorbs fast in periodontitis and is thus more difficult to graft. Marked differences exist with respect to tissue composition of the edentulous ridge between the maxilla and the mandible [14].

In 2021 Dorj, Lee et al. discovered that guided tissue regeneration treatment yields to better results in severe vertical bone loss in the molar region (class II furcations) in the mandible than in the maxilla [15]. Furthermore, stem cell properties of PDLSC isolated from third molars of maxilla and mandible show intrinsic differences in proliferation and plasticity [16]. Aside from cellular variations between the upper and lower jaws, there are also distinctions in diseases that are more relevant or more characteristic for the mandible. Alveolar osteitis, an inflammation of the alveolar bone caused by tooth extraction, for example, occurs more frequently in the lower jaw [17].

Few studies focused on cell signaling in mechanically stimulated human PDLSC cells, none of which considered the intrinsic differences between mandible and maxilla PDLSC cells [18]. We hypothesized that kinome analysis measuring kinase activity in upper and lower jaw PDLSC cells should reflect the state of major gene regulatory networks determining the regenerative potential of PDLSC, thus explaining differences in clinical outcome.

**Figure 1:**
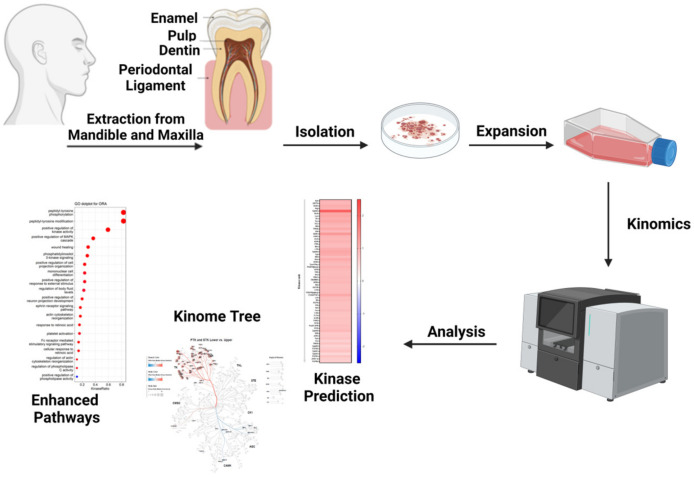
Schematic representation of the study design. Third molars of healthy donors were extracted from the upper and lower jaw of the same donors. Periodontal ligament was removed from the tooth, and periodontal ligament stem cells were isolated and expanded before being analyzed via kinomics technology. Created with BioRender.com., accessed on 14 February 2024.

Here we use kinase activity in PDLSC from mandible and maxilla to apply the gained knowledge to stem cell-mediated repair and tissue regeneration. For that, we used kinomics (Figure 1) as a key method to identify different pathways in PDLSC from upper and lower jaw isolated from the same individual (Ø age: 19.5). It is worth noting that paired periodontal ligament stem cells (PDLSC) were collected from both the maxilla and mandible of each donor, effectively eliminating patient-specific differences. This approach allowed us to focus specifically on the differences between the maxilla and mandible in terms of molecular regenerative properties. Kinases play critical roles in signal transduction which determines major cellular processes. Even though protein kinase genes constitute only 2% of the genome in most eukaryotes, protein kinases phosphorylate more than 30% of the cellular proteins [19]. Kinases are arranged in cascades, which are normally initiated by different receptors and then passed through different downstream effectors like the PI3K/mTOR and RAS-RAF-MAPK-pathway, among others, to further transmit their signals [20]. We present data on the phosphorylation status of cellular proteins in upper and lower jaw PDLSC cells from healthy donors. Using network analysis we map differential kinase activity onto known gene regulatory networks to explain the observed cell-intrinsic differences in growth and regeneration of these cells.

## 2. Results

### 2.1. PDLSC Characterization

**Figure 2:**
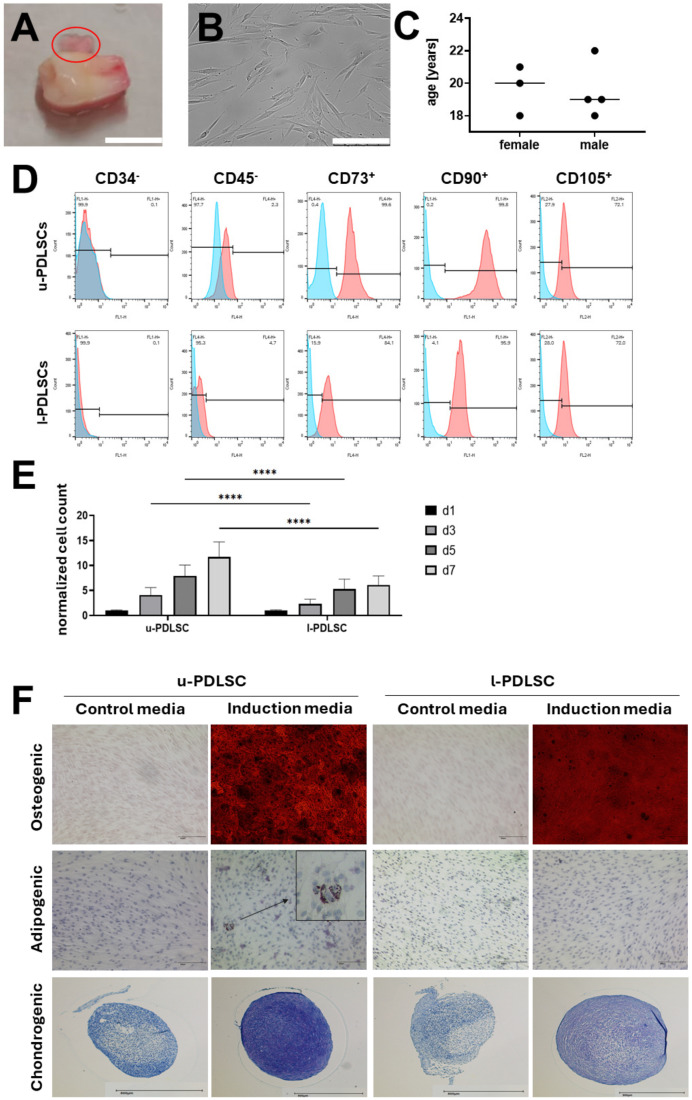
Characterization of PDLSC isolated from maxilla (u-PDLSC, u for upper jaw) and mandible (l-PDLSC, l for lower jaw). Periodontal tissue was scratched of third molars from healthy patients, and cells were isolated from mandible and maxilla and cultured separately ((**A**), scale bar: 1 cm, red circle indicates Periodontal Ligament). Cells from both origins showed fibroblastic morphology ((**B**), scale bar: 250 µm). A total of seven patients were integrated into this study, three female and four male patients with an average age of 20 and 19, respectively (**C**). Exemplary flow cytometric analysis of one donor u-PDLSC and l-PDLSC for stem cell markers (CD73^+^, CD90^+^, CD105^+^) and hematopoietic stem cell and endothelial cell surface markers (CD34^−^, CD45^−^ (**D**)). Normalized cell count of u-PDLSC and l-PDLSC after 1, 3, 5, and 7 days of culture was measured. PDLSC from maxilla show significantly higher proliferation after three days in culture, compared to PDLSC isolated from mandible (*p* < 0.0001) (**E**). Osteogenic, adipogenic (arrow indicates lipid vacuoles), and chondrogenic differentiation were performed for 21 days (**F**). u-PDLSC, periodontal ligament stem cells from upper jaw; l-PDLSC, periodontal ligament stem cells from lower jaw.

The International Society for Cellular Therapy defined fundamental protocols for mesenchymal stem/stromal cell (MSC) identification, which include detection of cell adhesion on tissue culture plastic, the presence or absence of specific surface antigens, and in vitro multilineage differentiation towards adipocytes, chondrocytes, and osteoblasts. This differentiation is tested by stimulating MSC with a cell differentiation media, followed by histological confirmation of the existence of differentiated cells. These so-called minimal criteria were used to characterize cells from periodontal tissue as stem cells. First, periodontal ligament tissue was isolated from the root of third molars of healthy donors from maxilla and mandible (Figure 2A). After scratching of the tissue from the tooth root, digestion with collagenase I was performed and isolated cells showed fibroblast-like morphology in culture (Figure 2B). Samples of three female (ø 20 years) and four male (ø 19 years) patients were collected for medical reasons at the authors’ dental facility (Figure 2C). To examine the characteristics of cultured PDLSC isolated from maxilla (u-PDLSC, u for upper jaw) and mandible (l-PDLSC, l for lower jaw) of the same donors, their cell surface antigen expression was investigated using flow cytometry. PDLSC tested negative for the hematopoietic stem cell and endothelial cell markers CD34^−^ and CD45^−^ and positive for CD73^+^, CD90^+^ and CD105^+^ (Figure 2D). Analysis of proliferation revealed significant differences between u-PDLSC and l-PDLSC on days three, five, and seven, confirming our previous study (Figure 2E) [16]. Multilineage potential was tested by differentiating PDLSC into osteoblasts, adipocytes, and chondroblasts for 21 days (Figure 2F). Alizarin Red staining following osteogenic for 21 days differentiation revealed more robust mineralization of u-PDLSC compared to l-PDLSC. Successful adipogenic differentiation was confirmed via Oil Red O staining. Lipid vacuoles could first be observed on day 21 in u-PDLSC, while no lipid vacuoles were detected in l-PDLSC. Compared to the non-differentiation cells, chondrogenic differentiation of both cell types caused more intense proteoglycan staining with Toluidin Blue. Cell pellets of chondrogenic differentiated PDLSC were more stable than pellets from undifferentiated PDLSC. Maxilla u-PDLSC had stronger proteoglycan staining than mandible l-PDLSC, suggesting that maxilla PDLSC possess greater chondrogenic differentiation potential than mandible PDLSC.

### 2.2. Phosphorylation of PTK in the Upper Jaw Was Promoted More Widely and Specifically than That of STK-Peptides

**Figure 3:**
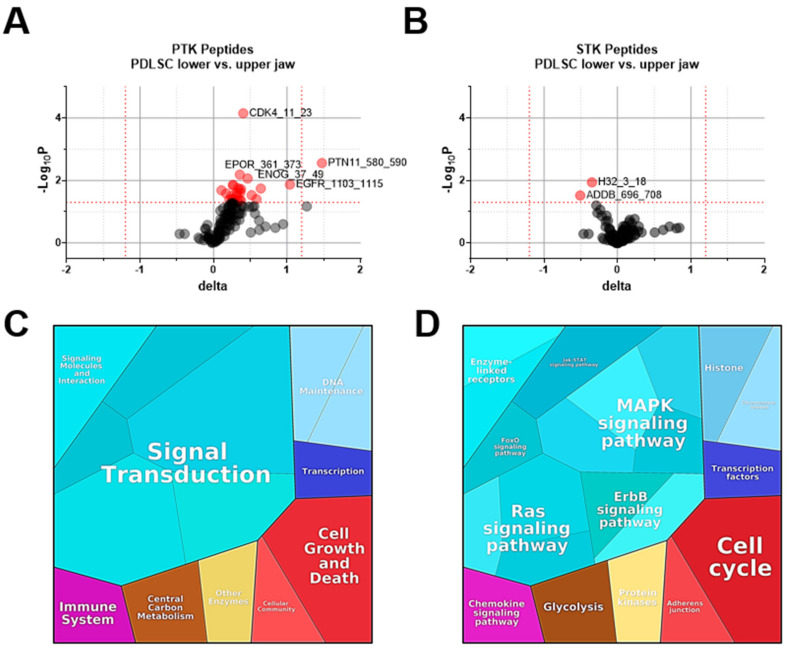
(**A**,**B**) Volcano plots of differentially phosphorylated PTK and STK peptides in arrays exposed to PDLSC lysates from maxilla and mandible. X−axis shows the effect size delta, and Y−axis shows the significance (−Log_10_ *p*-value). Red spots are peptides that are significantly different in phosphorylation between the two groups (*p* < 0.05, unpaired *t*-test). (**C**,**D**) Proteomap illustrating the corresponding category of significant peptides (combined PTKs and STKs) and the functions and pathways. Each peptide is shown by a polygon, and functionally related proteins are arranged in common regions. Area size of each polygon represents the median final score in PDLSC variant.

To determine protein kinase activity profiles of PDLSC from maxilla and mandible, we applied PamChip^®^ technology (Hertogenbosch, Netherlands) to analyze cells from seven donors. Protein tyrosine kinase PTK analysis revealed that 139 out of 196 PTK-specific peptides were phosphorylated, and of those, 19.42% (27/139) showed a significant difference between u-PDLSC and l-PDLSC (Figure 3A). In total, 89 out of 144 serine threonine kinase STK-specific peptides were phosphorylated, and 2.23% (2/89) were differentially phosphorylated in u-PDLSC and l-PDLSC (Figure 3B). The phosphorylation of PTK-peptides was enhanced more broadly than that of STK, although a couple of STKs’ activity increased considerably. Table 1 contains a detailed listing of peptide sequences contributing most to the significant changes between u- and l-PDLSC. The highest statistical significance in PTK phosphorylation were obtained for CDK4_11_23 (Uniprot Accession P11803) representing Cyclin-dependent kinase 4, a protein kinase complex regulating cell cycle G1 phase progression.

Significantly phosphorylated peptides (PTK and STK combined) were used to generate a proteomap (https://bionic-vis.biologie.uni-greifswald.de, accessed on 10 January 2024) using Uniprot Accession. Each peptide is shown by a polygon and functionally related proteins are grouped. Area size of each polygon represents the median final score in PDLSC variant between mandible and maxilla. Corresponding proteomap categories are presented in Figure 3C,D. Most differences between u-PDLSC and l-PDLSC related to signal transduction regulating cell growth, survival, and DNA maintenance. Phosphorylated peptides represented mitogen-activated protein kinase (MAPK), Ras, ErbB, Janus kinase-Signal Transducers and Activators of Transcription (Jak-STAT), and forkhead box O (FoxO) signaling pathways.

### 2.3. Two Ephrin Receptors Ranked among the Most Active Kinases

**Figure 4:**
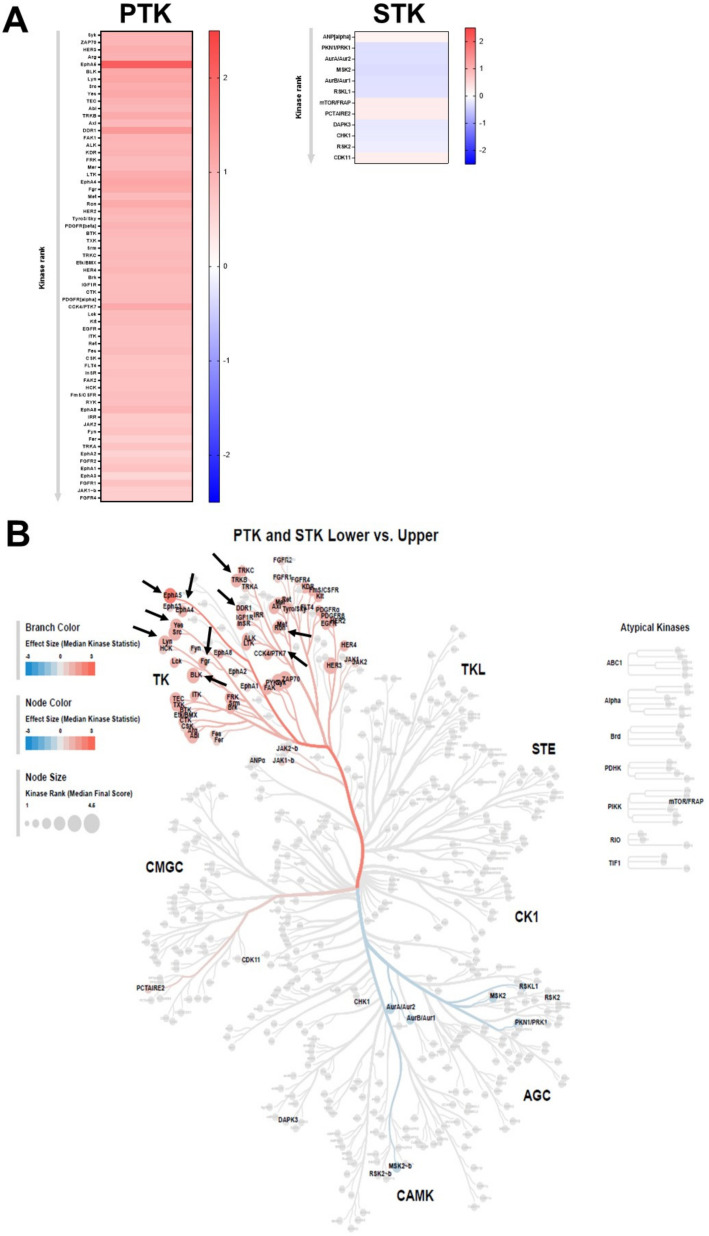
Significantly regulated kinases. (**A**) Heatmap of kinases that are upregulated upon PDLSC from the upper jaw compared to the lower jaw of seven donors. Red color reflects increased activity, while-blue color reflects decreased activity leading to reduced phosphorylation of the peptides. (**B**) Combined STK and PTK kinome tree. Activity of the kinases are represented on phylogenetic tree of the human protein kinase family. Dot size indicates specificity score and color denotes kinases statistic (Lower vs. Upper). Top 10 kinases for PTK are marked with an arrow. PTK = phosphotyrosine kinase, STK = serine-threonine kinase.

To identify the kinases mediating the observed phosphorylation patterns of the PamChip^®^ peptides (Hertogenbosch, Netherlands) the Upstream Kinase Analysis tool was applied. The predicted kinases were scored based on their significance in terms of the set of peptides used for the corresponding kinase. Figure 4 shows that out of 73 kinases predicted to mediate PTK phosphorylation, 64 showed significant differences in activity between the groups. As for STK, 92 kinases were predicted and 12 of them were significantly differentially expressed in u-PDLSC and l-PDLSC. The significant PTKs were all upregulated, while most of the STKs were downregulated. The kinases that had a significantly different activity in the maxilla compared to the mandible were visualized on the Coral kinome tree plot. The mean kinase statistics values, encoded in branch color node color, indicate the overall change of the peptide set that represents the kinase, with a value > 0 indicating kinase upregulation of PDLSC from mandible compared to maxilla (Figure 4B). Specific enhancement of PTK was observed in the Tyrosine Kinase (TK) families of the human kinome. The group of TK phosphorylates almost exclusively on tyrosine residues, as opposed to most other kinases that are selective for serine or threonine. It functions particularly as the relay of extracellular signals into the cell: over half of TKs are cell surface receptors (receptor tyrosine kinases, RTK), and many of the others function close to the surface of the cell. Upregulated STKs were part of the CMGC families, while downregulated ones are part of Calcium and Calmodulin-regulated kinases (CAMK) and AGC branch. The CMGC group includes key kinases: the MAPK growth- and stress-response kinases, the cell cycle CDK (cyclin dependent kinases), and kinases involved in splicing and metabolic control. This kinase family regulates the progression through the different phases of the cell cycle in association with their activating partners cyclins. Activated CAMK are involved in the phosphorylation of transcription factors and, therefore, in the regulation of expression of responding genes.

Top ten of PTK upstream kinases and their corresponding functions are listed in Table 2. The highest mean kinase statistic was predicted for Ephrin type-A receptor 5 (EphA5), followed by Epithelial discoidin domain-containing receptor 1 (DDR1), Lyn, Ephrin type-A receptor 4 (EphA4) and Yes-kinases. Amongst the top five predicted kinases two Ephrin receptors were listed as differentially active suggesting that Ephrin signaling may contribute to the differential growth pattern observed in u-PDLSC and l-PDLSC. Ephrin-A1–6 has been shown to preferentially bind glucose-phosphate-isomerase-linked Eph receptors numbered EphA1–8. Eph receptors are also known to signal through various different pathways and molecules, including small GTPases of the Rho and Ras family, focal adhesion kinase (FAK), the Jak/Stat pathway, and the PI3K pathway. In addition to regulating axonal guidance and synaptic plasticity, the ephrins/Ephs have been shown to serve broad non-neuronal functions, for example in vascular development, tissue-border formation, cell migration, and bone patterning [21,22].

### 2.4. Regulatory Network Analysis

**Figure 5:**
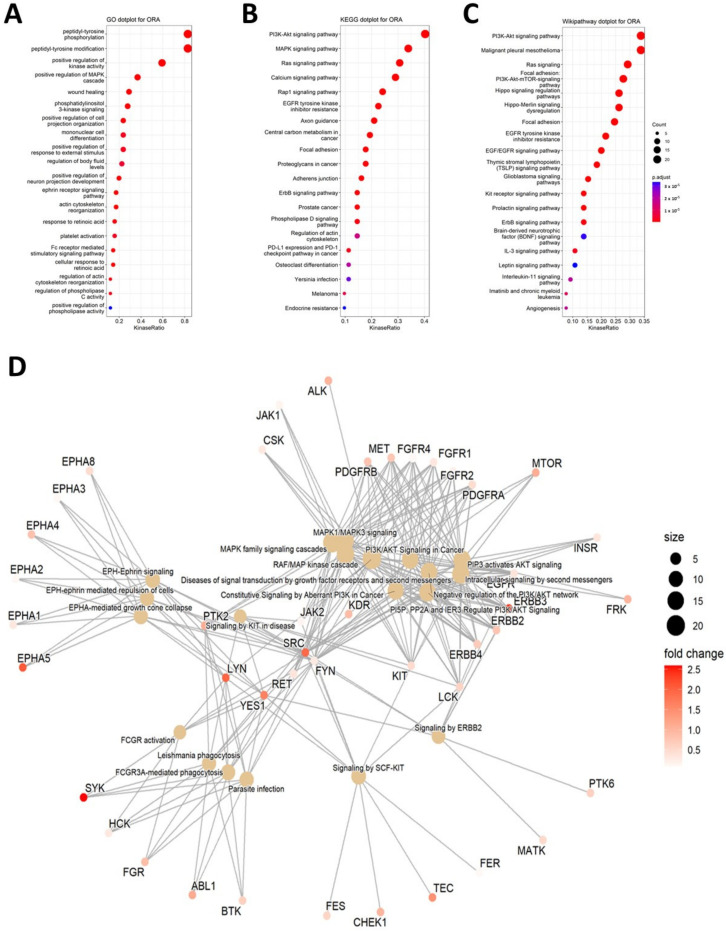
Dot plot for overrepresentation analysis (ORA) based on different databases, analysis with Gene Ontology (GO, (**A**)), Kyoto Encyclopedia of Genes and Genomes (KEGG, (**B**)) and Wikipathways (**C**). Results are ranked according to kinase ration, and dot size represents involved numbers/counts of kinases. Enriched pathways are visualized as network plot (**D**). Dot size represents the number of protein-encoding genes enriched in each pathway. Red color represents upregulated protein levels. White color represent downregulated protein levels, and dot size represents involved kinases.

To gain mechanistic insights into regulatory pathways underlying differential u-PDLSC and l-PDLSC, we performed overrepresentation analysis (ORA) which interrogates kinase sets associated with particular biological. For this purpose, three data bases were included, namely the Gene Ontology (GO, Figure 5A), Kyoto Encyclopedia of Genes and Genomes (KEGG, Figure 5B) and Wikipathways (Figure 5C). Figure 5D visualizes enriched pathways as a network plot.

The top five candidates of each data base are presented in Table 3. The MAPK signaling pathway and the PI3K pathway were ranked highest, together with the Ras signaling pathway and Calcium signaling pathway. Mitogen-activated protein kinase modules containing three sequentially activated protein kinases are key components of a series of vital signal transduction pathways that regulate processes such as cell proliferation, cell differentiation, and cell death [23]. Based on GO, the top overrepresented differences in pathways or function between mandible and maxilla are: peptidyl-tyrosine phosphorylation (Kinase Ratio: 63/76), peptidyl-tyrosine modification (Kinase Ratio: 63/76; pathway consists of 76 kinases of which 63 are significantly different), protein autophosphorylation (Kinase Ratio: 49/76), positive regulation of kinase activity (Kinase Ratio: 45/76), and positive regulation of MAPK cascade (Kinase Ratio: 28/76). Kinase Kyoto Encyclopedia of Genes and Genomes revealed significant differences of kinase upregulation in the upper jaw for: PI3K-Akt signaling pathway (Kinase Ratio: 25/62), MAPK signaling pathway (Kinase Ratio: 21/62), Ras signaling pathway (Kinase Ratio: 19/62), Calcium signaling pathway (Kinase Ratio: 18/62), and Rap1 signaling pathway (Kinase Ratio: 15/62). The last pathway database, named Wikipathways, showed significances for: PI3K-Akt signaling pathway (Kinase Ratio: 22/65), Malignant pleural mesothelioma (Kinase Ratio:22/65), Ras signaling (Kinase Ratio: 19/65), Focal adhesion: PI3K-Akt-mTOR-signaling pathway (Kinase Ratio: 18/65), and Hippo signaling regulation pathways (Kinase Ratio: 17/65).

Based on GO, top five pathways are related to peptidyl-tyrosine, phosphorylation, and kinase-regulation in general. These results underline the phosphorylation results of PTK. Other databases revealed significant differences in PI3K-Akt signaling pathway.

## 3. Discussion

Since mesenchymal stem cells were discovered more than 40 years ago, the relevance of stem cell research has become more important due the stem cells’ plasticity and patient-specific transplantations. These physiological and developmental characteristics make stem cells an integral part in the field of Regenerative Medicine due to their ability to generate entire tissues and organs from just a handful of progenitor cells [24]. Despite these similarities, different sources of MSC exhibit differences associated with the tissue origin from which they are derived. Dental stem cells are more likely to be suited for regeneration in dentistry if these source-dependent properties are taken into account. It is also crucial to note that dental tissue samples can be obtained easily during minimally invasive treatments [25].

Dental pulp stem cells (DPSC) were the first dental MSC-like cells which were identified and well-characterized [26]. Other tissue sources for stem cell isolation that have been revealed include periodontal ligament [27], dental germ [28], apical papilla [29], gingiva [30], and alveolar bone [31]. The periodontal ligament is a fibrous connective tissue made up of specialized cells that occurs between the bone-like cementum and the inner wall of the alveolar bone socket. Recently, several studies [26] identified a population of stem cells from human periodontal ligament (PDLSC) capable of differentiating along mesenchymal cell lineages to produce cementoblast-like cells, adipocytes, and connective tissue rich in collagen I in vitro and in vivo [27,32,33,34]. Pre-clinical research, including various in vivo models, as well as pilot studies and clinical trials reported thus far, encouraging findings in terms of the efficiency of PDLSC in periodontal complex regeneration and the safety of their usage in humans [35,36,37]. PDLSC have been intensively investigated over the years for these reasons, and they have shown potential in the regeneration of not just the periodontal complex, but also other dental and non-dental tissues [27,38].

However, concerning the differences between mandible and maxilla, the exact differences in pathways and kinase regulation are unknown. The purpose of this study was to characterize the isolated PDLSC from upper and lower jaw, as stem cells and comprehensively compare the kinase expression by using a novel approach to unravel pathways that may be involved and differentially regulated in periodontal wound healing velocity and efficacy in mandible and maxilla. We restricted ourselves to young and healthy donors (Ø age: 19.5) who had had their maxillary and mandibular third molars extracted at once.

In our study, we chose to utilize wisdom teeth for several reasons. Firstly, their frequent extraction due to issues like impaction or crowding ensured a readily available pool of samples for our research. Additionally, the larger size of wisdom teeth provided us with ample tissue and cells necessary for our investigations. Moreover, the advanced developmental stage of wisdom teeth, typically erupting later in life, allowed us to gain insights into various developmental phases of tooth biology, which was pertinent to our study objectives. Lastly, the relatively minimized disruption caused by wisdom tooth extraction in the oral cavity compared to other teeth extractions helped minimize discomfort and complications for our study participants, ensuring their safety and well-being throughout the research process. Overall, the utilization of wisdom teeth in our study was strategic and aligned with our research goals, providing a robust foundation for advancing our understanding of dental biology.

To provide the best possible comparison, u-PDLSC and l-PDLSC were treated using the identical methods and cultured independently. The two most frequent reasons for tooth loss are dental caries and periodontal disease [39]. Among the elderly population, periodontal disease is the most common ailment. This disease causes an inflammatory response in the periodontal tissues, and has both local and systemic effects through inflammatory cytokines [40]. Steroid hormones, such as sex hormones (oestradiol and testosterone) and glucocorticoids, are integral to bone development and remodeling processes. Hormonal imbalances, particularly the significant decline in sex hormones among aging individuals and postmenopausal women, can disrupt the equilibrium between osteoblastogenesis and osteoclastogenesis, leading to a loss of bone mass and strength. Studies indicate that insufficient levels of estrogen and androgen can detrimentally impact the proliferation and differentiation of bone marrow-derived mesenchymal stem cells (BMMSCs), as evidenced by diminished clonogenic assay outcomes, reduced mineral nodule formation, and decreased expression of osteogenic markers [41,42,43]. We were able to minimize the mentioned intra- and inter-subject variability by including only seven young and healthy donors in the study. But it is worth mentioning that not all factors, such as menstruation of female participants, can be eliminated.

Multipotent mesenchymal stromal cells express CD73^+^, CD90^+^, and CD105^+^ while lacking CD34^−^ and CD45^−^ expression, according to the International Society for Cellular Therapy (ISCT) [44]. The characterized PDLSC fulfilled these criteria. When comparing the proliferation rate of u-PDLSC and l-PDLSC it was shown that u-PDLSC have a significantly higher proliferation, regardless of the culture conditions. These results are in line with our previous findings [16]. Differentiation capacity towards osteoblasts, adipocytes, and chondrocytes were tested to ensure stem cell-like behavior. Alizarin red staining of PDLSC from mandible and maxilla after 21 days in culture revealed increased amount of calcium deposition, indicating a successful osteoblastic differentiation. Small lipid vacuoles were stained using Oil Red O staining to visualize adipogenic differentiation. These vacuoles can only be seen for u-PDLSC, but not for l-PDLSC. However, as compared to other MSC sources, PDLSC have a lower adipogenic differentiation capacity, which was already shown in other studies [16,45,46]. Our results also suggested that u-PDLSC have a better chondrogenic differentiation capability than l-PDLSC according to Toluidine blue staining of proteoglycans after 21 days. Zhang et al. already showed the chondrogenic differentiation potential of PDLSC in 2006, and also other studies could underline this result [47,48]. Hower the origin, upper or lower jaw, was not addressed. Our results showed that this is an important factor in terms of differentiation capability.

Kinomic activity of u-PDLSC and l-PDLSC was measured using PamChip^®^ peptide tyrosine kinase microarray system, which contains 196 unique phospho-site(s), which are peptide sequences obtained from Tyrosine kinase substrates and PamChip^®^ Ser/Thr Kinase assay. Each STK-PamChip^®^ array has 144 unique phospho-site(s), which are peptide sequences obtained from Ser/Thr kinase substrates. The phosphorylation of PTK-peptides was enhanced more widely and specifically than that of STK, although a couple of STKs’ activity increased considerably. In general, the PTK and STK-activity was higher in the upper jaw than in the lower jaw. Based on the phosphorylation, the identification of the top kinases responsible for it were identified using Upstream Kinases Analysis tool. Results showed that, amongst the top five predicted kinases, two Ephrin receptors were listed.

The human erythropoietin-producing hepatocellular (Eph) receptors include 14 type I transmembrane proteins, comprising the largest family of receptor tyrosine kinases (RTKs) [49], and are recognized to control a variety of cell-to-cell communication processes involved in tissue patterning during embryonic development and various different pathways and molecules, including small GTPases of the Rho and Ras family, focal adhesion kinase (FAK), the Jak/Stat pathway, and the PI3K pathway. [22,50,51,52]. Additionally, these receptors control a variety of specialized cellular processes, including insulin secretion, bone remodeling, epithelial homeostasis, synaptic plasticity, and immunological and inflammatory responses [51,53]. Based on sequence homology and binding affinity to their ligands, Eph receptors are divided into two subgroups, called EphAs (nine receptors) or EphBs (five receptors) [54]. It is consistent with their participation in stem cell activities during development and in adult tissue homeostasis that Eph receptors and ephrin ligands are expressed during embryogenesis, tissue homeostasis, and regeneration [55,56]. The system’s high complexity and plasticity are also related to the fact that Eph/ephrin signaling modulates numerous pathways, some of which are particularly important for cytoskeleton and cell adhesion modulation (cell attachment/detachment, migration, positioning, polarity, and cell shape), while others regulate gene transcription [57]. MSC generated from the stromal fraction of bone marrow (BM-MSC) and umbilical cord blood have been shown to express Eph and ephrins [58].

In 2022, Chen et al. performed single cell sequencing of periodontal tissue and detected the Ephrin–Eph signaling that has rarely been reported in periodontology before. He stated that Ephrin–Eph interactions may play distinct roles in modulating the process of periodontitis and assumed that abundance of Ephrin–Eph interactions in periodontal tissues from patients with severe chronic periodontitis after treatment has a significant influence on osteogenesis. An inhibition of this interaction may facilitate clinical bone defect healing after initial periodontal therapy [59]. In particular, EphA5 was identified as an inhibitor in long-term cultures that led to the deterioration of BM-MSC differentiation capacity. Therefore, EphA5 may be a negative regulator of BM-MSC osteogenic differentiation [60]. In our study, EphA5 was ranked the top kinase (Mean Kinase Statistic: 2.106), indicating a higher kinase expression in the upper jaw compared to the lower jaw. Together with recent findings, that PDLSC from mandible have a lower osteogenic differentiation capacity than from the maxilla, this study may explain the regulatory factors involved in this process.

Differently regulated pathways between maxilla compared to the mandible, revealed that PI3K-Akt signaling pathway was ranked top one based on KEGG and Wikipathways. This pathway is a critical intracellular signal transduction pathway related to several fundamental cellular processes, including cell motility, growth, apoptosis, and differentiation [61,62]. A growing amount of research suggests that PI3K and its downstream effectors, such as Akt, play a role in the control of bone development and formation [63]. Kratchmarova and Blagoev et al. discovered that the PI3K pathway is activated in MSC after treatment with plated-derived growth factor (PDGF) and resulted in suppressed osteogenic differentiation [64]. Another study showed that dexamethasone can suppress osteogenesis of osteoblast via the PI3K-Akt pathway in vitro and in vivo [65].

To the best of our knowledge, this work is the first to examine in vitro PDLSC kinase activity in PDLSC from maxillary and mandibular molars. We assume that u-PDLSC and l-PDLSC have distinct inherent characteristics and that differences in kinase level are involved in significant regulatory pathways. Additional research in this field of periodontal remodeling is required to better understand the molecular mechanisms underlying periodontal remodeling and the differences between PDLSC derived from the maxilla and mandible. In particular, in vivo studies to examine the potential in PDLSC regenerative potential when transplanted into recipient tissue are required.

## 4. Materials and Methods

### 4.1. Cell Isolation and Expansion

Human third molars were taken from seven healthy patients, four male and three female, and were divided into maxillary and mandibular groups based on their origin. To achieve maximum comparability, only teeth and tissue from individuals with both maxillary and mandibular third molar extraction were included in the present study. Isolation was performed, as previously described [16].

Periodontal ligament was scraped from the middle third of the root under sterile conditions and digested in a solution of 1% collagenase type 1 (Worthington, Lakewood, USA) for 1 h at 37 °C. The resulting cell suspension was centrifuged for 5 min at 500× *g* before the supernatant was discarded. The cell pellet was then resuspended in culture medium before being seeded in a T-75 culture flask (Cellstar, Greiner Bio-One, Kremsmünster, Austria). Isolated PDLSC were cultured in PDLSC expansion medium containing Dulbecco’s Modified Eagle Medium (DMEM), high-glucose (Fisher Scientific, Schwerte, Germany), 10% fetal calf serum (FCS; Pan-Biotech, Aidenbach Germany), 50 mg/L L-ascorbic-acid (Sigma-Aldrich, Taufkirchen, Germany), 100 IU/mL penicillin (Fisher Scientific, Schwerte, Germany), and 100 μg/mL streptomycin (Fisher Scientific, Schwerte, Germany). Strict donor and origin separation (upper jaw-PDLSC [u-PDLSC] or lower jaw-PDLSC [l-PDLSC]) was maintained. Collection and usage of PDLSC cells from discarded patient biomaterial were approved by the ethics committee of the University Clinics of RWTH Aachen, Germany (approval number EK 374/19), and all experiments were carried out in accordance with the relevant guidelines and regulations.

### 4.2. Flow Cytometry

Flow cytometry was used to examine u-PDLSC and l-PDLSC for specific MSC-related surface epitopes. Cells from third passage were trypsinized, counted, and stored in flow cytometry buffer (0.09% FCS in phosphate-buffered saline (PBS; Fisher Scientific, Schwerte, Germany)). Cells were centrifuged at 500× *g* for 5 min at 4 °C before being resuspended in 100 µL flow cytometry buffer containing the antibodies. The APC-, PE-, and FITC-isotype controls were diluted with flow cytometry buffer to achieve concentrations of 0.2 µg/100 µL, 0.2 µg/100 µL, and 0.5 µg/100 µL, respectively. Conjugated antibodies against CD34, CD45, CD73, CD90, and CD105 (eBioscience, Frankfurt on the Main, Germany) were diluted in flow cytometry buffer at concentrations of 0.5 µg/100 µL, 0.06 µg/100 µL, 0.124 µg/100 µL, 1 µg/100 µL, and 1 µg/100 µL, respectively. The cells were then cultured for 30 min at 4 °C before being centrifuged for 5 min at 500× *g* and the supernatant was collected. Finally, cells were resuspended in 300 µL flow cytometry buffer. A FACS Canto II cytometer (BD Bioscience, Heidelberg, Germany) was used to analyze immunophenotypes, and at least 10.000 events were recorded for each donor.

### 4.3. Cell Proliferation

Periodontal ligament stem cells from the upper and lower jaw were seeded in a density of 10.000 cells/well in 12-well culture plates to determine the proliferation rate. Cell counts were taken after one, three, five, and seven days of culture in matching medium using a CASY cell counter (Roche Innovatis, Munich, Germany). All measurements were done in triplicate.

### 4.4. Osteogenic, Adipogenic and Chondrogenic Differentiation

Cells were seeded in 24-well culture plates in a density of 10.000/cm^2^ for osteogenic differentiation. The osteogenic induction medium consists of DMEM low glucose (Fisher Scientific, Schwerte, Germany), 10% FCS, 100 nM dexamethasone, 10 mM sodium-glycerophosphate, and 0.05 mM L-ascorbic acid (all from Sigma-Aldrich, Taufkirchen, Germany). Three times per week, a medium exchange was performed.

For adipogenic differentiation, cells were seeded at 25.000/cm^2^ in 24-well culture plates. Medium was changed twice per week, alternating between induction and maintenance medium. Adipogenic induction medium (AIM) consisted of DMEM high glucose, 10% FCS, 1 mM dexamethasone, 0.2 mM indomethacin, 0.5 mM 3-isobutyl-1-methylxanthine, and 0.01 mg/mL insulin (all from Sigma-Aldrich, Taufkirchen, Germany), while adipogenic maintenance medium (AMM) consisted of DMEM high glucose, 10% FCS, and 0.01 mg/mL insulin.

For chondrogenic differentiation, pellet culture with 250.000 cells per 15 mL polypropylene tube was performed. Cells were centrifuged at 500× *g* for 5 min. After that, the pellets were grown in serum-free chondrogenic induction media, which included DMEM high glucose, 5% ITS Plus Premix (BD Biosciences, Heidelberg, USA), 100 nM dexamethasone, 0.17 mM L-ascorbic acid, 100 µg/mL sodium pyruvate, and 40 µg/mL L-proline (all Sigma-Aldrich, Taufkirchen, Germany). When the medium was changed, 10 ng/mL TGF-ß3 (Thermo Fisher Scientific, Waltham, USA) was added. Medium was changed three times per week. PDLSC media without additives was used as a control.

### 4.5. Alizarin Red, Oil Red O and Toluidine Blue Staining

Alizarin Red staining was used to visualize calcium-rich deposits developed during osteogenic differentiation. The cells were fixed in ice-cold 70% ethanol (−20 °C) for one hour and stained with 40 mM Alizarin Red S (Sigma-Aldrich, Taufkirchen, Germany) for 10 min. PBS was used to wash away unbound Alizarin Red, and microscopic images were recorded.

The lipid vacuoles generated during adipogenic differentiation were visualized using Oil Red O staining. The Oil Red O solution was produced according to the manufacturer’s instructions (Sigma-Aldrich, Taufkirchen, Germany). Cells were fixed for 30 min in cold 50% ethanol (4 °C) and stained for 10 min in Oil Red O solution. Haematoxylin was used to counterstain the nuclei of the cells.

Pellets of chondrogenically differentiated cells were fixed in 4% formaldehyde overnight and paraffin-embedded for Toluidine blue staining. A microtome was used to cut 2 µm sections. Slices were stained with 1% Toluidine blue (Sigma-Aldrich, Taufkirchen, Germany) in 0.1 M sodium acetate buffer after deparaffinization with xylene and rehydration to visualize proteoglycans of stem cell-derived chondrocytes.

### 4.6. Peptide Tyrosine Kinase Activity Profiling

PamChip^®^ peptide tyrosine kinase microarray system on PamStation^®^12 (PTK; PamGene International, Hertogenbosch, Netherlands) was used to determine tyrosine kinase profiles. Each PTK-PamChip^®^ array includes 196 unique phospho-site(s), which are peptide sequences obtained from Tyrosine kinase substrates. Each peptide on the chip forms a 15-amino acid sequence that serves as a tyrosine kinase substrate and represents a possible endogenous phosphorylation site. Peptide phosphorylation is observed by detecting the fluorescent signal given by binding the FITC-conjugated PY20 anti-phosphotyrosine antibody. Human PDLSC of maxilla and mandible were seeded in 6-well plates in passage two until they reached a confluency of 80%. Cells were washed with ice-cold PBS and lysed for 15 min on ice with M-PER Mammalian Extraction Buffer (1:100 each; Thermo Fischer Scientific, Waltham, USA) with Halt Phosphatase Inhibitor and EDTA-free Halt Protease Inhibitor Cocktail. In a pre-cooled centrifuge, lysates were centrifuged for 15 min at 16.000× *g* at 4 °C and supernatant was collected. Protein quantification was carried out using the PierceTM Coomassie Plus (Bradford) Assay (Thermo Fischer Scientific, Waltham, USA) according to manufacturers’ instructions. For PTK assay, 10 µg of protein was applied per array and the standard Pamgene method was followed using the provided reagents. To create the PTK Basic Mix, the freshly frozen lysate was initially mixed with 4 µL of 10× protein PTK reaction buffer (PK), 0.4 µL of 100× bovine serum albumin (BSA), 0.4 mL of 1 M dithiothreitol (DTT) solution, 4 µL of 10× PTK additive, 4 µL of 4 mM ATP and 0.6 µL of monoclonal anti-phosphotyrosine FITC-conjugate detection antibody (clone PY20). By adding distilled water (H20), the total volume of the PTK Basic Mix was adjusted to 40 µL. Before loading the PTK Basic Mix to the array, a blocking step with 30 µL of 2% BSA was performed for each array and washing with PTK solution for PamChip^®^ preprocessing was conducted. After that, 40 µL of PTK Basic Mix was added to each PamChips^®^ array, and microarray tests were then performed for 94 cycles. A CCD camera PamStation^®^12 captured an image at kinetic read cycles 32–93 at 10, 50, and 200 ms, as well as at end-level read cycles 10, 20, 50, 100, and 200 ms. Using the BioNavigator software version 6.3 (PamGene International, Hertogenbosch, Netherlands), the spot intensity at each time point was assessed (and compensated for local background). Upstream Kinase Analysis (UKA) [66], a functional scoring approach (PamGene), was used to rank kinases based on combined specificity and sensitivity scores.

### 4.7. Serine/Threonine Kinase Activity Profiling

The PamChip^®^ Ser/Thr Kinase assay (STK; PamGene International, Hertogenbosch, Netherlands) was used to evaluate serine/threonine kinase profiles. Each STK-PamChip^®^ array has 144 unique phospho-site(s), which are peptide sequences obtained from Ser/Thr kinase substrates. In passage two, human PDLSC isolated from the maxilla and mandible were seeded in 6-well plates until they achieved 80% confluency. Cells were rinsed with ice-cold PBS before being lysed for 15 min on ice using M-PER Mammalian Extraction Buffer (1:100; Thermo Fischer Scientific, Waltham, MA, USA) with Halt Phosphatase Inhibitor and EDTA-free Halt Protease Inhibitor Cocktail. Lysates were centrifuged for 15 min at 16.000× *g* at 4 °C in a pre-cooled centrifuge, and supernatant was collected. Protein quantification was performed using the PierceTM Coomassie Plus (Bradford) Assay (Thermo Fischer Scientific, Waltham, MA, USA), according to instructions by the manufacturer. For the STK assay, 2.0 µg of protein and 400 µM ATP were applied per array together with an antibody mixture to detect phosphorylated Ser/Thr. A second FITC-conjugated antibody is used to detect the phosphorylation signal after an hour (30 °C) incubation, in which the sample is pushed back and forth through the porous material to enhance binding kinetics and decrease assay time. The imaging was done with an LED imaging device, and the spot intensity at each time point was measured (and adjusted for local background) using the BioNavigator software version 6.3 (PamGene International, Hertogenbosch, Netherlands). Upstream Kinase Analysis (UKA) [66], a functional scoring approach (PamGene), was used to rank kinases based on combined specificity scores (based on peptides associated with a kinase gathered from 6 databases) and sensitivity ratings.

### 4.8. Prediction of Upstream Kinases and Pathway Analysis

Phosphopeptides that passed the quality control were mapped for predicted upstream kinases. Permutation analysis resulted in a specificity score (mapping of peptides to kinases) and a significance score (difference between gene variant carriers and control subjects) for each kinase. Based on the combined scores ((specificity + significance) = median final score), an arbitrary threshold of 1.2 was applied. The median final score was used to rank and predict top kinase hits, which were different between the two study groups. The differentially regulated upstream peptides were mapped using Proteomaps (https://bionic-vis.biologie.uni-greifswald.de, accessed on 14 February 2024 [67]). The area size of each polygon represents the median final score in variants between PDLSC isolated from maxilla and mandible. Peptides being significantly and differently phosphorylated between PDLSC from the upper and lower jaw were used for possible pathways and networks study. Over-representation analysis (ORA) of the Kyoto Encyclopedia of Genes and Genomes (KEGG) database for the kinases with significant differences were performed using the ClusterProfiler 4.0 R-package [68]. The R package disease ontology semantic and enrichment analysis (DOSE) [69] was utilized to analyze the biological complexities of the kinases and how they correlate with multiple annotation categories. The interactions were visualized in a network plot with the R package Reactome Pathway Analysis (ReactomePA) v1.44.0 [70]. The *p*-values were adjusted for multiple comparisons by false discovery rate (FDR).

### 4.9. Statistics

Data are expressed as mean ± standard error of the mean (SEM). Statistical analysis was performed using GraphPad Prism version 9.1.1 (GraphPad Software, Inc., San Diego, CA, USA). Significance was tested using either Student’s *t*-test (with Welch correction as required) or Mann–Whitney U-test for normally and non-normally distributed data, unless stated otherwise. A two-tailed *p*-value < 0.05 was considered statistically significant.

## 5. Conclusions

Periodontal ligament stem cells were isolated from seven healthy donors and characterized as stem cells via surface epitopes and multilineage differentiation capacity towards osteoblasts, adipocytes, and chondroblasts. PamGene^®^ technology was used to identify differences in kinase regulation between PDLSC from mandible and maxilla. It is important to highlight that paired periodontal ligament stem cells were obtained from both the maxilla and mandible of every donor, seven in total. This method effectively mitigated patient-specific variations, enabling us to concentrate exclusively on discerning the distinctions between the maxilla and mandible concerning molecular regenerative properties. Our findings showed that PDLSC from the upper jaw have a significant higher proliferation rate and better differentiation capability. Upstream kinase analysis revealed two EphA receptors that are significantly higher expressed in the mandible compared to maxilla. These receptors, especially EphA4, are known to work as inhibitors for osteogenic differentiation. Pathway analysis showed significant differences in the regulation of PI3K-Akt pathway in the lower jaw compared to the upper jaw. A few studies stated the involvement of PI3K pathway in the suppression of osteogenesis. This might be a first hint on unraveling the molecular mechanism in differentiation efficacy of PDLSC from upper and lower jaw. This study underlined the importance of location when isolating PDLSC.

Our findings have important implications for clinical applications and regenerative medicine. By elucidating differences in proliferation rate, differentiation capability, and kinase activity between PDLSCs from the upper and lower jaw, our study opens up new possibilities for tailoring regenerative therapies to specific anatomical locations. For instance, understanding the molecular mechanisms underlying enhanced osteogenic differentiation in PDLSCs from the upper jaw could inform the development of targeted therapies for bone regeneration in craniofacial reconstruction procedures.

Furthermore, our identification of EphA receptors and the PI3K-Akt pathway as potential key regulators of differentiation efficacy in PDLSCs underscores the importance of location-specific factors in stem cell behavior. This knowledge can guide the development of more effective strategies for manipulating PDLSCs in tissue engineering and regenerative medicine applications.

In summary, our study not only advances our understanding of PDLSC biology, but also offers valuable insights into the potential clinical applications of PDLSC-based therapies. We believe that our findings pave the way for further research aimed at harnessing the therapeutic potential of PDLSCs for various regenerative medicine applications.

## Figures and Tables

**Table 1:** Significant Phosphorylations of PTK and STK-Peptides.

PTK				
ID	Uniprot Accession	Sequence	Delta	−log_10_P
CDK4_11_23	P11802	EIGVGAYGTVYKA	0.405	4.150
PTN11_580_590	Q06124	SARVYENVGLM	1.476	2.558
EPOR_361_373	P19235	SEHAQDTYLVLDK	0.358	2.189
ENOG_37_49	P09104	SGASTGIYEALEL	0.463	2.067
EGFR_1103_1115	P00533	GSVQNPVYHNQPL	1.039	1.872
PDPK1_369_381	O15530	DEDCYGNYDNLLS	0.263	1.860
LCK_387_399	P06239	RLIEDNEYTAREG	0.269	1.842
PAXI_24_36	P49023	FLSEETPYSYPTG	0.333	1.754
PGFRB_1002_1014	P09619	LDTSSVLYTAVQP	0.644	1.743
PGFRB_768_780	P09619	SSNYMAPYDNYVP	0.375	1.724
MAPK3_198_210_C203S	Q16644	ALQTPSYTPYYVA	0.353	1.709
GSK3B_210_222_C218S	P49841	GEPNVSYISSRYY	0.108	1.685
LYN_391_403	P07948	VIEDNEYTAREGA	0.315	1.616
P85A_600_612	P27986	NENTEDQYSLVED	0.365	1.613
FAK1_569_581	Q05397	RYMEDSTYYKASK	0.171	1.587
EPHA7_607_619	Q15375	TYIDPETYEDPNR	0.324	1.547
EPOR_419_431	P19235	ASAASFEYTILDP	0.519	1.531
MK12_180_189_M182B	P53778	SEBTGYVVTR	0.242	1.507
PTN6_558_570	P29350	KHKEDVYENLHTK	0.253	1.411
EGFR_1190_1202	P00533	STAENAEYLRVAP	0.584	1.407
PRRX2_202_214	Q99811	WTASSPYSTVPPY	0.266	1.404
RET_1022_1034	P07949	TPSDSLIYDDGLS	0.357	1.380
ZAP70_313_325	P43403	SVYESPYSDPEEL	0.329	1.368
CDK2_8_20	P24941	EKIGEGTYGVVYK	0.278	1.361
ANXA1_14_26	P04083	IENEEQEYVQTVK	0.374	1.358
VGFR2_1168_1180	P35968	AQQDGKDYIVLPI	0.322	1.307
STK				
ID	Uniprot Accession	Sequence	Delta	−log_10_P
H32_3_18	Q71DI3	RTKQTARKSTGGKAPR	−0.347	1.941
ADDB_696_708	P35612	GSPSKSPSKKKKK	−0.507	1.521

**Table 2:** Top 10 PTK predicted upstream kinases.

PTK			
Kinase Name	Kinase Uniprot ID	Mean Kinase Statistic	Function
Ephrin type-A receptor 5 (EphA5)	P54756	2.106	Contact-dependent bidirectional signaling into neighboring cells.
Epithelial discoidin domain-containing receptor 1 (DDR1)	Q08345	1.334	Cell surface receptor for fibrillar collagen and regulates cell attachment to the extracellular matrix, remodeling of the extracellular matrix, cell migration, differentiation, survival and cell proliferation.
Lyn	P07948	1.165	Regulation of innate and adaptive immune responses, hematopoiesis, responses to growth factors and cytokines, and integrin signaling.
Ephrin type-A receptor 4 (EphA4)	P54764	1.141	Contact-dependent bidirectional signaling into neighboring cells.
Yes	P07947	1.128	Regulation of cell growth and survival, apoptosis, cell–cell adhesion, cytoskeleton remodeling, and differentiation.
tyrosine-protein kinase 7 (CCK4/PTK7)	Q13308	1.121	Cell adhesion, cell migration, cell polarity, proliferation, actin cytoskeleton reorganization, and apoptosis.
B lymphocyte kinase (BLK)	P51451	1.113	Involved in B-lymphocyte development, differentiation, and signaling.
Ron	Q04912	1.102	Transduces signals from the extracellular matrix into the cytoplasm.
Tropomyosin receptor kinase B (TRKB)	Q16620	1.092	Transmembrane receptor protein; has been found to play a pivotal role in neural development.
Fgr	P09769	1.091	Regulation of immune responses.

**Table 3:** Top 5 pathways that are differently regulated between maxilla compared to the mandible. Overrepresentation analysis (ORA) based on GO, KEGG, Wikipathways, and enriched pathways.

Ranking	Pathway	*p*-Value	*p*-adj
GO			
1	peptidyl-tyrosine phosphorylation	5.523 × 10^−96^	1.29 × 10^−92^
2	peptidyl-tyrosine modification	9.54 × 10^−96^	1.29 × 10^−92^
3	protein autophosphorylation	1.10 × 10^−76^	9.93 × 10^−74^
4	positive regulation of kinase activity	8.25 × 10^−53^	5.58 × 10^−50^
5	positive regulation of MAPK cascade	2.06 × 10^−25^	5.77 × 10^−23^
KEGG			
1	PI3K-Akt signaling pathway	1.45 × 10^−18^	2.17 × 10^−16^
2	MAPK signaling pathway	1.31 × 10^−15^	6.54 × 10^−14^
3	Ras signaling pathway	3.22 × 10^−15^	1.21 × 10^−13^
4	Calcium signaling pathway	9.24 × 10^−14^	2.44 × 10^−12^
5	Rap1 signaling pathway	3.02 × 10^−11^	6.49 × 10^−10^
Wikipathways			
1	PI3K-Akt signaling pathway	1.86 × 10^−14^	7.26 × 10^−13^
2	Malignant pleural mesothelioma	3.70 × 10^−12^	1.24 × 10^−10^
3	Ras signaling	2.92 × 10^−16^	2.27 × 10^−14^
4	Focal adhesion: PI3K-Akt-mTOR-signaling pathway	3.38 × 10^−11^	8.79 × 10^−10^
5	Hippo signaling regulation pathways	1.64 × 10^−18^	3.84 × 10^−16^
Enriched pathways			
1	Diseases of signal transduction by growth factor receptors and second messengers	1.59 × 10^−13^	8.07 × 10^−12^
2	RAF/MAP kinase cascade	1.01 × 10^−13^	6.59 × 10^−12^
3	MAPK1/MAPK3 signaling	1.46 × 10^−13^	8.07 × 10^−12^
4	MAPK family signaling cascades	1.30 × 10^−12^	4.55 × 10^−11^
5	PIP3 activates AKT signaling	6.40 × 10^−13^	2.49 × 10^−11^

## Data Availability

The data that support the findings of this study are available from the corresponding author, H.M., upon reasonable request.
